# Mechanisms of synergistic suppression of ALK-positive lung cancer cell growth by the combination of ALK and SHP2 inhibitors

**DOI:** 10.1038/s41598-023-37006-2

**Published:** 2023-06-20

**Authors:** M. A. Berry, A. R. Bland, J. C. Ashton

**Affiliations:** Department of Pharmacology and Toxicology, School of Biomedical Sciences, University of Otago, Dunedin, New Zealand

**Keywords:** Cancer, Lung cancer, Non-small-cell lung cancer

## Abstract

Lung cancer is a major cause of cancer-related deaths. Alectinib is the first line of treatment for patients with ALK-positive lung cancer, but the survival rate beyond 2–3 years is low. Co-targeting secondary oncogenic drivers such as SHP2 is a potential strategy for improving drug efficacy. This is because SHP2 is expressed ubiquitously, but ALK expression is largely restricted to cancer cells. Thus, the combination of ALK and SHP2 inhibitors may provide a way to restrict synergistic cytotoxicity to cancer cells only, by reducing the dose of SHP2 inhibitors required for anticancer action and minimising SHP2-dependent systemic toxicity. The objective of this study was to investigate whether the combination of a SHP2 inhibitor (SHP099) with alectinib would synergistically suppress the growth of ALK-positive lung cancer cells. Our results demonstrated that the drug combination significantly and synergistically decreased cell viability at relatively low concentrations in ALK-positive H3122 and H2228 cells, due to G1 cell cycle arrest and increased apoptosis because of suppressed downstream RAS/MAPK signalling. The drug combination also induced the expression of mediators of the intrinsic apoptotic pathway, Bim and cleaved caspase-3, and modulated the expression of cell cycle mediators cyclin D1, cyclin B1, and phosphorylated CDK1.

## Introduction

Substantial advances have been made in recent years in the survival of patients with non-small cell lung cancer (NSCLC) expressing druggable receptor tyrosine kinase (RTK) mutations. The greatest success has been achieved with drugs selectively targeting over-expressed and/or mutated forms of epidermal growth factor receptor (EGFR), anaplastic lymphoma kinase receptor (ALK), ROS proto-oncogene 1 (ROS1), and KRAS proto-oncogene (KRAS)^1^. These targets are often responsive to drug monotherapy, however, adding to the list of druggable targets in NSCLC are secondary drivers; targets that have the most therapeutic potential when inhibited in combination with primary oncogenic drivers (such as those listed above)^2,3^.

The non-receptor protein tyrosine phosphatase, Src homology region 2-containing protein tyrosine phosphatase (SHP2), has been identified as one such potential target^4^. SHP2 acts downstream of numerous RTKs and mediates cell signalling, including the important mediator of cell proliferation and survival, RAS-ERK^5^. SHP2 was not considered to be druggable until 2012 when it was demonstrated that the SHP2 inhibitor II-B08, enhanced the efficacy of phosphoinositide 3-kinase (PI3K) inhibition in models of myeloproliferative disease^4^. SHP2 can now be targeted using a range of compounds, including several that have entered clinical trials^6^. Of these, SHP099 is an orally bioavailable small molecule allosteric inhibitor that promotes the inactive state of the protein^7^.

Although SHP099 has little activity against other specific enzyme systems that are most commonly associated with toxicity^8^, as SHP2 is expressed ubiquitously throughout the body^9^ the toxicity of SHP2 inhibitors poses potential risks^10^. A strategy that has proven to be effective for reducing toxicity for some drug combinations is to reduce the minimum effective dose (MED) of a drug by combining it with another drug/s to produce synergistic efficacy^11^. If this can be targeted to cancer cells, then this should not also cause synergistic toxicity. Using SHP2 inhibitors in combination with drugs targeting a primary oncogenic driver might be a way to harness the anti-cancer properties of SHP2 inhibitors whilst minimising toxicity. Given that SHP2 intermediates the growth and survival pathways of RTKs, oncogenic RTKs emerge as a potential co-treatment partner with SHP2 inhibitors^12^.

ALK is a promising candidate for co-targeting alongside SHP2 in the treatment of NSCLC^13^. EML4-ALK is a chromosomal rearrangement caused by fusion between echinoderm microtubule-associated protein-like 4 (EML4) and ALK, creating constitutively active ALK^14^. ALK-positive (ALK^+^) lung cancer accounts for ~ 6% of all NSCLC cases^15^. As ALK is not widely expressed after embryonic development is complete, ALK inhibitor off-target systemic toxicity is low^16^. ALK inhibitors beginning with crizotinib were first approved by the FDA in 2011^17^, with clinical trials quickly demonstrating the superiority of ALK inhibitors over chemotherapy for ALK^+^ NSCLC treatment^18^. Currently, the first-line ALK inhibitor is alectinib, which has superior blood–brain barrier penetration compared to crizotinib^15^. However, patient outcomes are limited by tumour resistance^19–21^.

If SHP2 inhibition combines synergistically with ALK inhibition to reduce tumour cell growth, then SHP2 inhibitors might improve outcomes with alectinib in ALK^+^ NSCLC at sub-toxic doses of SHP2 inhibition. Therefore, this study aimed to test the hypothesis that combining the SHP2 inhibitor, SHP099, with alectinib will result in synergistic ALK^+^ cell growth suppression in two different ALK^+^ NSCLC cell lines. The drug combination was then used to investigate the cellular mechanisms driving synergistic cell growth suppression.

## Methods

### Materials

Alectinib and SHP099 were purchased from LC Laboratories (Woburn, Massachusetts, USA) and MedChem Express (Monmouth Junction, NJ, USA), respectively. Roswell Park Memorial Institute (RPMI) 1640 medium and TrypLE were purchased from Life Technologies (Grand Island, NY, USA). Penicillin/streptomycin, sulforhodamine B (SRB), trichloroacetic acid (TCA), and dimethylsulfoxide (DMSO) were obtained from Sigma-Aldrich (St Louis, MO, USA). Fetal bovine serum (FBS) was purchased from Sigma-Aldrich (Auckland, NZ). Bovine serum albumin (BSA) was obtained from Life Technologies (Auckland, NZ). Bicinchoninic acid (BCA) protein assay reagent A was purchased from ThermoFisher Scientific (USA). SuperSignal West Pico Chemiluminescent and CL-XPosure film were obtained from ThermoFisher Scientific (Auckland, NZ). Acrylamide and precision plus protein kaleidoscope standard were purchased from Bio-Rad Laboratories (Hercules, CA, USA). FxCycle PI/RNase was purchased from Life Technologies (California, USA).

Primary antibodies against ALK (D5F3, 3633S), pALK (Tyr1640, 3341S), SHP2 (3752S), pSHP2 (Y542, 3751S), pERK (T202/Y204, 9101S), Bcl2 (3498S), Bim (2189S), Bax (5023S), cleaved caspase-3 (9664 T), p27 (2552S), cyclin D1 (2978S) and CDK4 (12790S) were purchased from Cell Signaling Technology (Danvers, MA, USA). pCDK1 (Thr14, Tyr15, 44-686G) and pCDK4 (Thr172, PA5-64,482) were purchased from Invitrogen (MA, USA). The ERK1/2 (9102S), CDK1 (HPA003387), and β-tubulin (T5293) antibodies were purchased from Sigma-Aldrich (St Louis, MO, USA). The secondary antibody horseradish peroxidase-conjugated goat anti-rabbit was purchased from Calbiochem (San Diego, CA, USA) and horseradish peroxidase-conjugated goat anti-mouse antibody was purchased from EMD Millipore Corp (USA).

### Cell culture

The H3122 human adenocarcinoma ALK^+^ NSCLC cell line harbouring the EML4-ALK variant one fusion gene was gifted by Professor Daniel Costa, Harvard University. Variant one of the EML4-ALK fusion is the most prevalent variant at 33% of EML4-ALK NSCLC cases^22^. The H2228 human adenocarcinoma ALK^+^ NSCLC cell line harbouring the EML4-ALK variant three fusion gene was purchased from ATCC (Virginia, USA). A549 human lung adenocarcinoma cell line harbouring a KRAS gene codon 12-point mutation were gifted from Dr Gregory Giles, University of Otago. All cells were stored in a humidified incubator with 5% CO_2_ at 37 °C. Cell lines were maintained in RPMI 1640 medium supplemented with 1% penicillin/streptomycin and either 2%, 5%, or 10% FBS for A549, H3122, and H2228 cells, respectively.

### Cell viability

H3122, H2228, and A549 cells were seeded at 7.0 × 10^3^, 2.5 × 10^4^, and 4.0 × 10^3^ cells/well, respectively, in 96-well plates for 24 h then treated with the respective drug treatment concentrations for a further 72 h. Cells were fixed in 10% TCA for 30 min, and cell viability was assessed using the SRB cell viability assay according to^23^. Results are presented as a percentage of the vehicle control, and the EC_50_ was determined using non-linear regression ((inhibitor) vs. response variable slope (four parameters)) in GraphPad Prism v8.0 from three independent experiments repeated in technical triplicate^24^.

### Flow cytometry

H3122 and H2228 cells were seeded at 3.0 × 10^5^ and 5.0 × 10^5^ cells/well, respectively, in 6 well plates for 24 h. Cells were treated with the EC_50_ values for the respective cell lines for an additional 24 h. H3122 cells were treated with the vehicle control (0.002% DMSO), alectinib (0.018 μM), SHP099 (11.8 μM), or a combination of both. H2228 cells were treated with the vehicle control (0.003% DMSO), alectinib (0.033 μM), SHP099 (15.9 μM), or the combination.

For cell cycle analysis, after the treatment period, the cells were washed with PBS, trypsinised, and centrifuged at 797 × *g* for 4 min, followed by fixation in 70% ethanol for at least 24 h. Cells were then washed with PBS, centrifuged at 449 × *g* for 10 min, resuspended in FxCycle PI/RNase staining solution, and incubated in the dark for 30 min. Samples were analysed using the FACs Canto II flow cytometer for H3122 and BD LSRFortessa flow cytometer for H2228 cells, and data were analysed using FlowJo LLC software (v10). Results were presented as a percentage of cells in each phase of the cell cycle from three independent experiments^25^.

### Western blotting

H3122 and H2228 cells were seeded at 1.0 × 10^6^ and 2.5 × 10^6^ cells per petri dish, respectively, and incubated for 24 h. Cells were treated with the EC_50_ values for the respective cell lines for an additional 24 h. H3122 cells were treated with the vehicle control (0.002% DMSO), alectinib (0.018 μM), SHP099 (11.8 μM), or a combination of both. H2228 cells were treated with the vehicle control (0.001% DMSO), alectinib (0.033 μM), SHP099 (15.9 μM), or a combination of both. Following the 24-h drug treatment, cells were lysed with lysis buffer (50 mM Tris base (pH–7.5), 150 mM NaCl, 1 mM EDTA, 1 mM EGTA, 0.5% NP-40, and 0.5% SDS) containing protease inhibitors (10 mM sodium fluoride, 1 mM sodium orthovanadate and 1 mM complete protease inhibitor mix) and stored at − 80 °C overnight. Cells were then sonicated, centrifuged at 20,854 × *g* for 8 min at 4 °C. 20 μg of samples were then loaded onto SDS-PAGE using a Mini Protean 3 system (Bio-Rad Laboratories, USA) at 120 V for 90 min. Proteins were transferred to an activated PVDF membrane at 100 V for 90 min. Membranes were blocked in 2% to 5% BSA for 1 h and then incubated overnight at 4 °C in the respective primary antibodies. The primary antibody dilutions used were; ALK rabbit monoclonal antibody (mAb) (1:1000), pALK rabbit antibody (1:1000), β-tubulin mouse mAb (1:2000), SHP2 rabbit polyclonal antibody (pAb) (1:1000), pSHP2 rabbit pAb (1:1000), ERK1/2 rabbit pAb (1:1000), pERK1/2 (T202/Y204) rabbit pAb (1:1000), Bim rabbit antibody (1:1000), Bcl2 rabbit mAb (1:1000), Bax rabbit mAb (1:1000), cyclin D1 rabbit mAb (1:1000), cyclin B1 rabbit mAb (1:1000), CDK1 rabbit pAb (1:1000), pCDK1 rabbit pAb (1:1000), CDK4 rabbit mAb (1:1000), pCDK4 (Thr172) rabbit pAb (1:1000), cleaved caspase-3 rabbit mAb (1:1000), p27 rabbit mAb (1:1000).

Following incubation, the membranes were probed with secondary antibodies; anti-mouse (1:1000) for β-tubulin and anti-rabbit (1:1000) for all other antibodies. SuperSignal West Pico Chemiluminescent substrate with CL-XPosure film was used to visualise the proteins of interest. A GS-710 calibrated imaging densitometer (Bio-Rad) with Quantity One software (v4.6.7) was used to quantify the density of the bands where the background of each band was subtracted, and the proteins normalised to the housekeeper protein β-tubulin. For each antibody, three biological replicates were performed^25^.

### Statistical analysis

Cell viability and Western blotting data were analysed using a one-way ANOVA followed by a Bonferroni post hoc test. Data are expressed as mean ± SEM. Data analyses were carried out using GraphPad Prism v8.0 (GraphPad, San Diego, USA), where a significant difference was reported when *p* < 0.05^26^.

To determine if the combination of alectinib and SHP099 exhibited synergy, the Bliss model^27^ was carried out on the cytotoxicity data to derive a combination index (CI) (Eq. 1) as described previously by Bland et al*.*^28^. The graphical form is presented as a ratio of the predicted Bliss CI to the actual observation of response for cell death. E_A_ is the effect of alectinib when applied alone, E_S_ is the effect of SHP099 when applied alone, and (E_A_ x E_S_) is the product of the effect of alectinib and SHP099. E_AS_ is the degree of the effect of alectinib and SHP099 when applied in combination. A CI of more than one indicates an inhibitory effect of the drugs, and a CI of less than one indicates positive synergy between the two drugs in combination. A CI of one indicates additivity with separate drug targets when in combination.

1 $$Bliss\;CI\; = \;\frac{{E_{A} + E_{S} - \left( {E_{A} \times E_{S} } \right)}}{{E_{AS} }}$$

Bliss combination equation for calculation of synergy when alectinib and SHP099 are applied in combination.

Flow cytometry data for both cell cycle and apoptosis were analysed using a two-way ANOVA followed by a Bonferroni post hoc test. Data are expressed as mean ± SEM. Data were gathered using FlowJo LLC software v10 and analysis was carried out using GraphPad Prism v8.0 (GraphPad, San Diego, USA). Significant differences were reported when *p* < 0.05.

## Results

### The effect of alectinib and SHP099 on signalling pathways in ALK^+^ lung cancer cells

**Figure 1:**
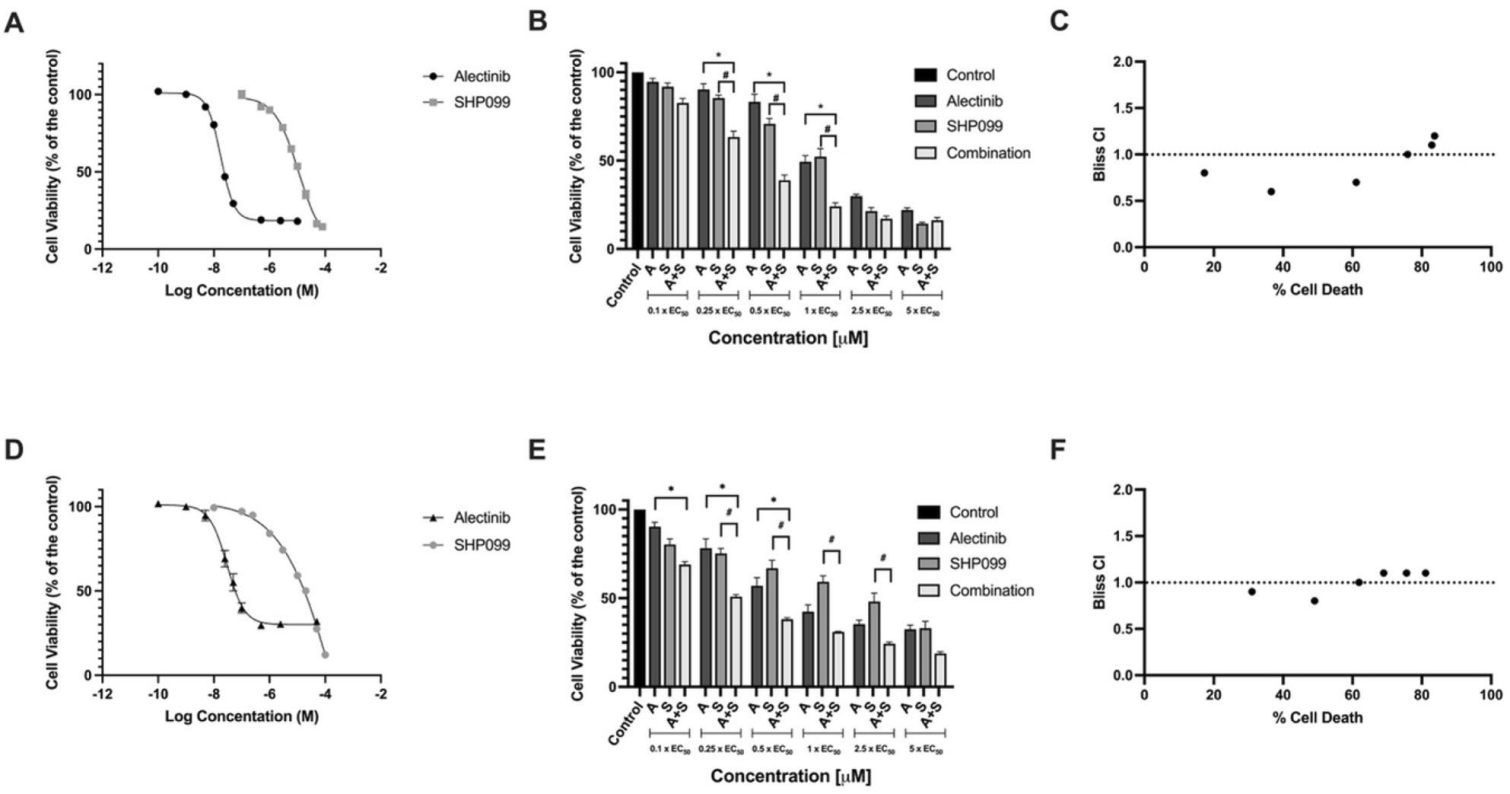
The effect of alectinib, SHP099 and their combination on cell viability of H3122 and H2228 cells. Effect of alectinib and SHP099 on cell viability in (**a**) H3122 and (**d**) H2228 cells. Data points are expressed as mean ± SEM. EC_50_ values were determined using non-linear regression analysis. Effects on cell viability in the presence of alectinib and SHP099 in combination in (**b**) H3122 and (**e**) H2228 cells, where A = alectinib, S = SHP099, A + S = alectinib and SHP099 in combination. Data points are expressed as mean ± SEM. * represents a significant difference for alectinib vs combination (*p* < 0.05) and # represents a SHP099 vs combination (*p* < 0.05). Combination indices (y-axis) for (**c**) H3122 and (**f**) H2228 cells calculated using the Bliss method for each of the combinations in “B” and “E” respectively. The x-axis represents the amount of cell death obtained by each combination. For all data n = 3 independent experiments performed in technical triplicate.

We first examined the relative potency of alectinib and SHP099 in ALK^+^ lung adenocarcinoma cells; H3122 (ALK^+^ variant 1) and H2228 (ALK^+^ variant 3) cells were highly sensitive to alectinib, with EC_50_ values of 0.018 μM and 0.033 μM, respectively (Fig. 1A, D). By comparison, for A549 cells (ALK^-^/KRAS^+^), we were unable to obtain EC_50_ values for alectinib as test concentrations were constrained by drug solubility (Supplementary Fig. S1). All three cell lines, H3122, H2228, *and* A549 were similarly sensitive to SHP099, though at significantly higher concentrations compared to alectinib (EC_50_ values of 11.8 μM, 15.9 μM, and 16.3 μM, respectively) (Fig. 1A, D, and Supplementary Fig. S1). These values were used to calculate EC_50_ equivalent concentrations that were used in subsequent experiments. I.e., concentrations of alectinib and SHP099 normalised to the EC_50_ concentration for a drug/cell line combination; for example, EC_50_ for SHP099 in H3122 cells is 11.8 μM, 0.5 × EC_50_ for the same drug/cell line combination is 5.9 μM, and so on.

In the H3122 cells, 0.25 × EC_50_, 0.5 × EC_50_, and 1 × EC_50_ concentrations of both alectinib and SHP099 decreased cell viability in combination significantly more than when applied alone (Fig. 1B) with the results for the three lowest concentrations tested displaying evidence of synergy (Fig. 1C). Similar decreases in cell viability were observed in the H2228 cells, with two of the lower concentrations (0.25 × EC_50_ and 0.5 × EC_50_) significantly decreasing the cell viability in the combination compared to either drug alone (Fig. 1E). The two lowest concentrations produced synergistic suppression of cell growth, with the third having an additive effect (Fig. 1F).

### The effect of alectinib and SHP099 on signalling pathways in ALK^+^ cancer cells

**Figure 2:**
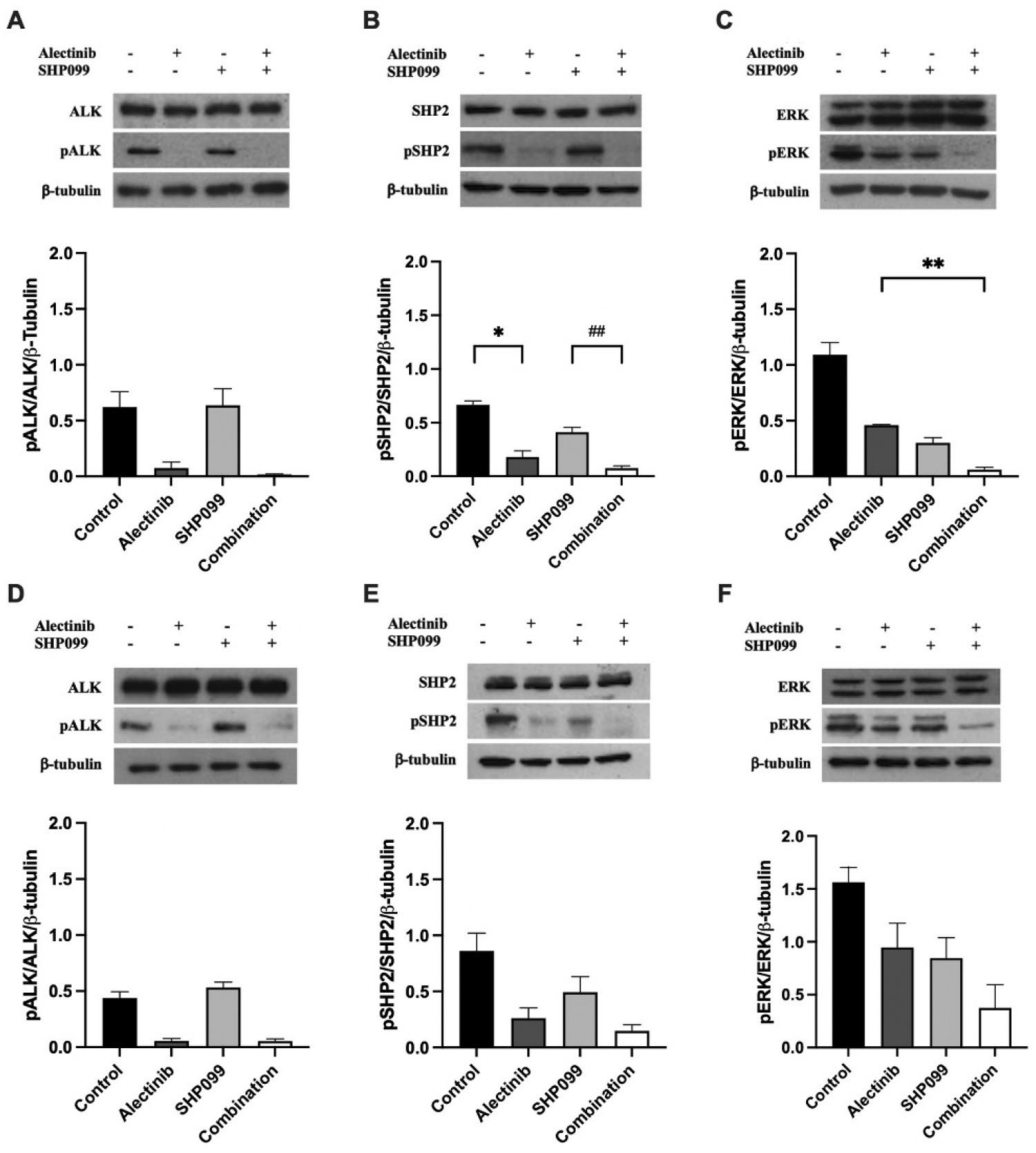
The effect of alectinib, SHP099, and the combination on downstream signalling pathways at EC_50_ concentrations in H3122 and H2228 cells. Representative and quantified Western blots in H3122 cells for (**a**) pALK/ALK, (**b**) pSHP2/SHP2 and, (**c**) pERK/ERK, and β-tubulin following 24 h treatment. Representative and quantified Western blots in H2228 cells for (**d**) pALK/ALK, (**e**) pSHP2/SHP2 and, (**f**) pERK/ERK, and β-tubulin following 24 h treatment. For all Western blots, data are expressed as mean ± SEM and were analysed using a one-way ANOVA followed by Bonferroni post hoc test. *represents a significant difference for control versus alectinib, *p* < 0.05, **represents a significant difference for alectinib vs combination, *p* < 0.01 and ## represents a significant difference for SHP099 vs combination, *p* < 0.01. n = 3 independent experiments.

We next investigated the biochemical mechanisms by which the drug combination (alectinib with SHP099) reduced the growth of ALK^+^ cancer cells using Western blotting. Our results demonstrated that the addition of SHP099 did not significantly alter the phosphorylation (i.e., activation) of ALK in either cell line tested (*p* > 0.05, Fig. 2A, D). Alectinib alone inhibited the phosphorylation of SHP2, and the combination of SHP099 and alectinib significantly reduced phosphorylation to a greater extent than SHP099 alone in H3122 (*p* < 0.05, Fig. 2B) but not H2228 cells (*p* > 0.05, Fig. 2E). However, Western blotting for the phosphorylation ERK, the major downstream intracellular signalling mediator of ALK and SHP2, in the H3122 cell line demonstrated a strong effect from combining the two drugs (87% decrease, *p* < 0.001 for alectinib compared with the combination; Fig. 2C).

In the H3122 cells, after a 24-h treatment, alectinib and the combination of alectinib and SHP099 resulted in a significant decrease (*p* < 0.001) in pALK/ALK levels of 0.88- and 0.97-fold respectively compared to the control (*p* < 0.05) but no further decrease in pALK/ALK levels with the combination compared to alectinib alone (*p* > 0.05). There was no change in protein levels with individual SHP099 treatment alone (*p* > 0.05, Fig. 2A). A similar trend was observed in the H2228 cells, with the addition of SHP099 to alectinib not resulting in a statistically significant reduction in cell growth compared with alectinib alone (*p* > 0.05, Fig. 2D).

For H3122 SHP2 activation (pSHP2/SHP2 expression) there was a significant (0.38-fold, *p* < 0.05) decrease in protein levels following SHP099 treatment compared to the control, with a similar decrease following alectinib treatment (0.73-fold, *p* < 0.001) and a very strong decrease following treatment with the combination compared with the control (0.89-fold, *p* < 0.0001, Fig. 2B). By contrast, there were no significant changes in H2228 cells in pSHP2/SHP2 levels (*p* > 0.05, Fig. 2E) following SHP099 treatment alone compared to the control, while there was an approximately 0.70-fold decrease in pSHP2/SHP2 expression following alectinib treatment alone, and a similar (0.83-fold) decrease for the combination, compared to the control (*p* < 0.05).

Inhibition of downstream signalling as indicated by decreased pERK/ERK expression was observed for both cell lines. In the H3122 cells, there were approximate 0.58-, 0.73-, and 0.95-fold decreases in pERK/ERK expression compared to the control following alectinib alone, SHP099 alone, and the combination respectively (*p* < 0.001, Fig. 2C). For the H2228 cells, the only significant change in protein expression occurred following the combination treatment (0.67-fold reduction compared to the control, *p* < 0.05) with there being no significant differences between any of the monotherapies and combination (*p* > 0.05, Fig. 2F).

### The effect of the combination of alectinib and SHP099 on the cell cycle in ALK^+^ NSCLC cells

To characterise the mode of cell growth suppression by the combination of alectinib with SHP099 more precisely we assayed its effects first on the cell cycle using flow cytometry, and then on cell cycle mediators using Western blotting.

**Figure 3:**
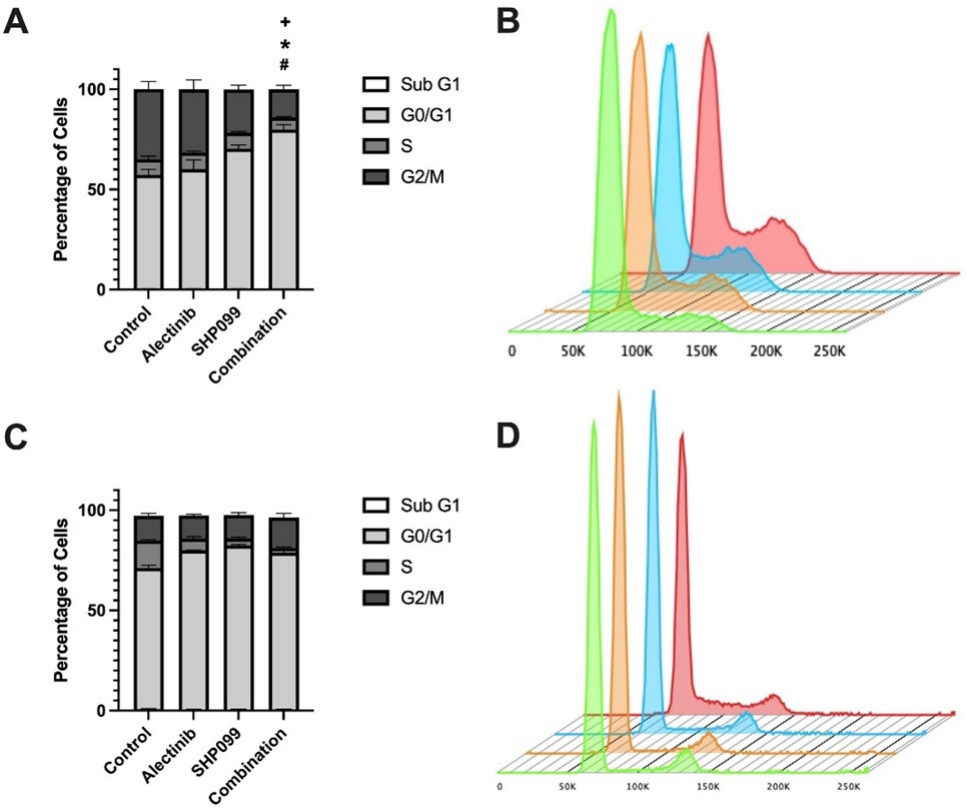
The effect of alectinib, SHP099 and the combination on cell cycle arrest at EC_50_ concentrations in H3122 and H2228 cells following 24-h treatment. Percentage of cells in each phase of the cell cycle for (**A**) H3122 and (**C**) H2228 cells. Data are expressed as mean ± SEM and were analysed using a two-way ANOVA followed by Bonferroni post-hoc test. * indicates significance for alectinib vs combination for G0/G1 and + for G2/M, p < 0.05. # indicates significance for SHP099 versus combination. Representative flow cytometry cell cycle histogram for (**B**) H3122 and (**D**) H2228 cells. Differently shaded histograms denote different treatments (from front to back: combination, SHP099, alectinib, control). n = 3 independent experiments.

In the H3122 cells, changes in cell number were detected in both the G0/G1 and G2/M phases following 24-h drug treatment. There was a significant increase in the number of cells in the G0/G1 phase for SHP099 and combination treatment groups by 13% and 23% respectively, compared to the control (*p* < 0.05, Fig. 3A, B). When comparing the individual drug treatments of alectinib and SHP099 to the combination there was a significant increase in the number of cells in G0/G1 phase for combination group compared to either drug alone by 20% or 10% respectively (*p* < 0.05, Fig. 3A, B). In addition, there was an associated significant decrease of cells in the G2/M phase of the combination treatment by 18% compared to alectinib alone (*p* < 0.05, Fig. 3A, B).

Interestingly, in the H2228 cells, the changes occurred in the G0/G1 and S phases of the cell cycle. When compared to the control, there was a significant increase of cells in the G0/G1 phase for alectinib (9%), SHP099 (11.5%), and the combination (7.8%) (*p* < 0.05, Fig. 3C, D). The opposite occurred for the S phase, with a significant decrease occurring for alectinib (7.7%), SHP099 (10.2%) and the combination (11.3%) compared to the control (*p* < 0.05, Fig. 3C, D). However, unlike the H3122 cells, when compared to the combination, neither of the monotherapies had any significant changes in any phases of the cell cycle (*p* > 0.05, Fig. 3C, D).

**Figure 4:**
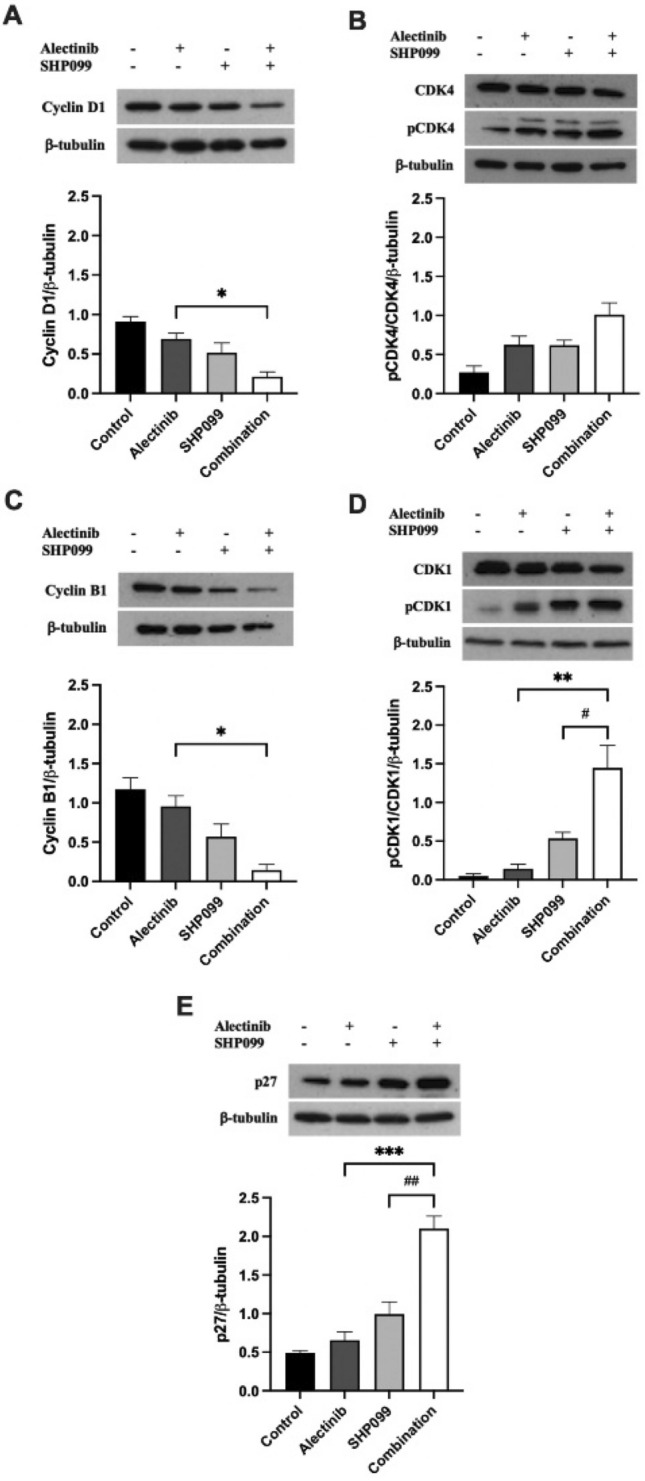
The effect of alectinib, SHP099, and the combination on cell cycle mediators at EC_50_ concentrations in H3122 cells. Representative and quantitative Western blots for (**a**) cyclin D1, (**b**) pCDK4/CDK4, (**c**) cyclin B1, and (**d**) pCDK1/CDK1, (**e**) p27, and the housekeeper protein β-tubulin after exposure to various drug treatments for 24 h. For all Western blots data are expressed as mean ± SEM and were analysed using a one-way ANOVA followed by Bonferroni post hoc test. *a significant difference for alectinib vs combination, *p* < 0.05, **represents a significant difference for alectinib vs combination, *p* < 0.01, # represents a significant difference for SHP099 vs combination, *p* < 0.05, *** represents a significant difference for alectinib versus combination, *p* < 0.001 and ## represents a significant difference for SHP099 vs combination, *p* < 0.01. n = 3 independent experiments.

**Figure 5:**
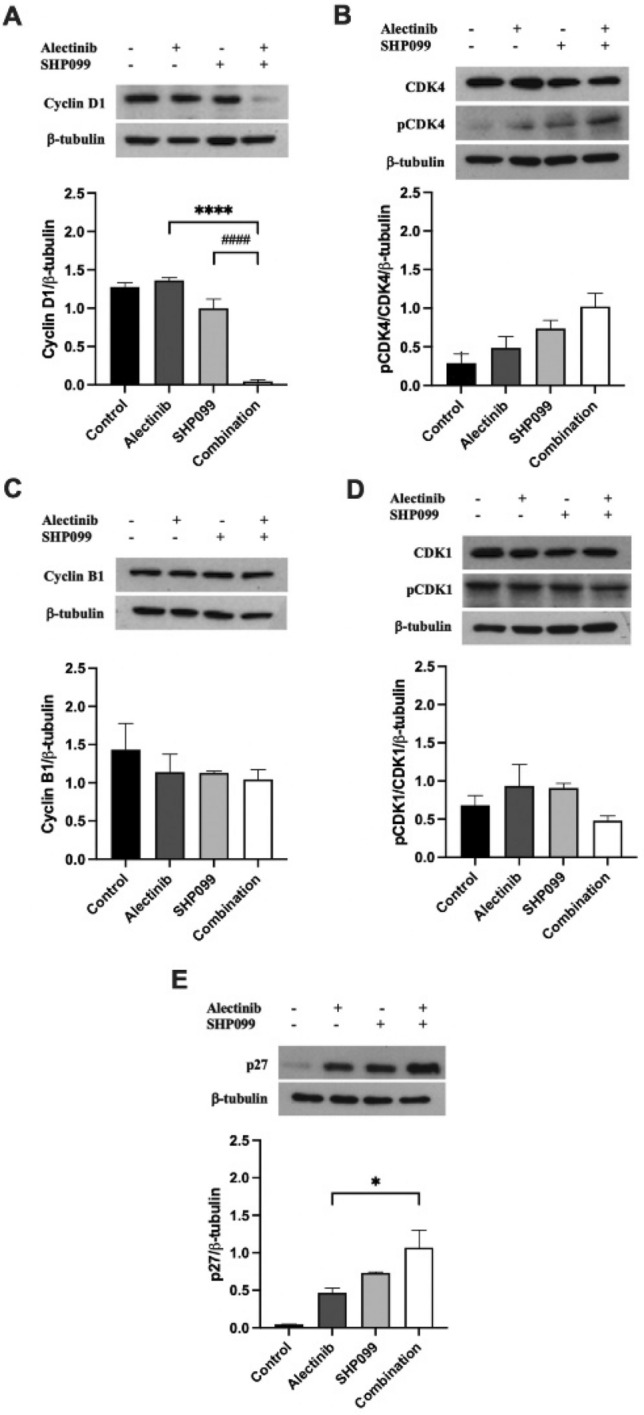
The effect of alectinib, SHP099, and the combination on the cell cycle at EC_50_ concentrations in H2228 cells. Representative and quantitative Western blots for (**a**) cyclin D1, (**b**) pCDK4/CDK4, (**c**) cyclin B1, and (**d**) pCDK1/CDK1, (**e**) p27, and the housekeeper protein β-tubulin after exposure to various drug treatments for 24 h. For all Western blots data are expressed as mean ± SEM and were analysed using a one-way ANOVA followed by Bonferroni post hoc test. ****represents a significant difference for alectinib vs combination, *p* < 0.0001 and ####represents a significant difference for SHP099 vs combination, p < 0.0001. n = 3 independent experiments.

Using Western blotting we detected a statistically significant 0.7-fold decrease in cyclin D1 expression in H3122 cells after 24-h treatment with the combination of alectinib and SHP099 compared with alectinib alone (*p* < 0.05, Fig. 4A) and a profound decrease in H2228 cells in the combination compared with either drug alone (0.97-fold and 0.95-fold respectively for alectinib and SHP099, *p* < 0.001, Fig. 5A). For both cell lines there was a non-significant trend upwards for phosphorylated CDK4 levels (*p* > 0.05, Fig. 4B, 5B). The expression of cyclin B1 was reduced 0.85-fold by the alectinib/SHP099 combination compared to alectinib alone in H3122 cells (*p* < 0.05, Fig. 4C) but not in H2228 cells (*p* > 0.05, Fig. 5C). In H3122 cells, the phosphorylation of CDK1 was profoundly increased in the monotherapies (2.1-fold for alectinib, 10.5-fold for SHP099) or combination (30.2-fold) compared to control (*p* < 0.001, Fig. 4D), in addition to the combination having a significant increase in expression levels compared to individual alectinib and SHP099 treatments (9.1-fold and 1.7-fold respectively, *p* < 0.05, Fig. 4D), an effect not detected in H2228 cells (Fig. 5D).

We also investigated the intermediary between growth factor receptor signalling and cell cycle mediator, p27, following 24-h exposure to EC_50_ concentrations of alectinib, SHP099, or the combination, again using Western blot. In H3122 cells there was a strong combination effect, with the drug combination increasing p27 expression to a greater extent than either drug alone (2.2-fold increase from alectinib, *p* < 0.001, and 1.1-fold increase from SHP099, *p* < 0.01, Fig. 4E). For H2228 cells there was a similar trend toward increasing p27 with drug treatments, particularly for the combination, which was significantly greater than alectinib alone (12.9-fold increase, *p* < 0.01, Fig. 5E).

### The effect of the combination of alectinib and SHP099 on apoptosis in ALK^+^ NSCLC cells

**Figure 6:**
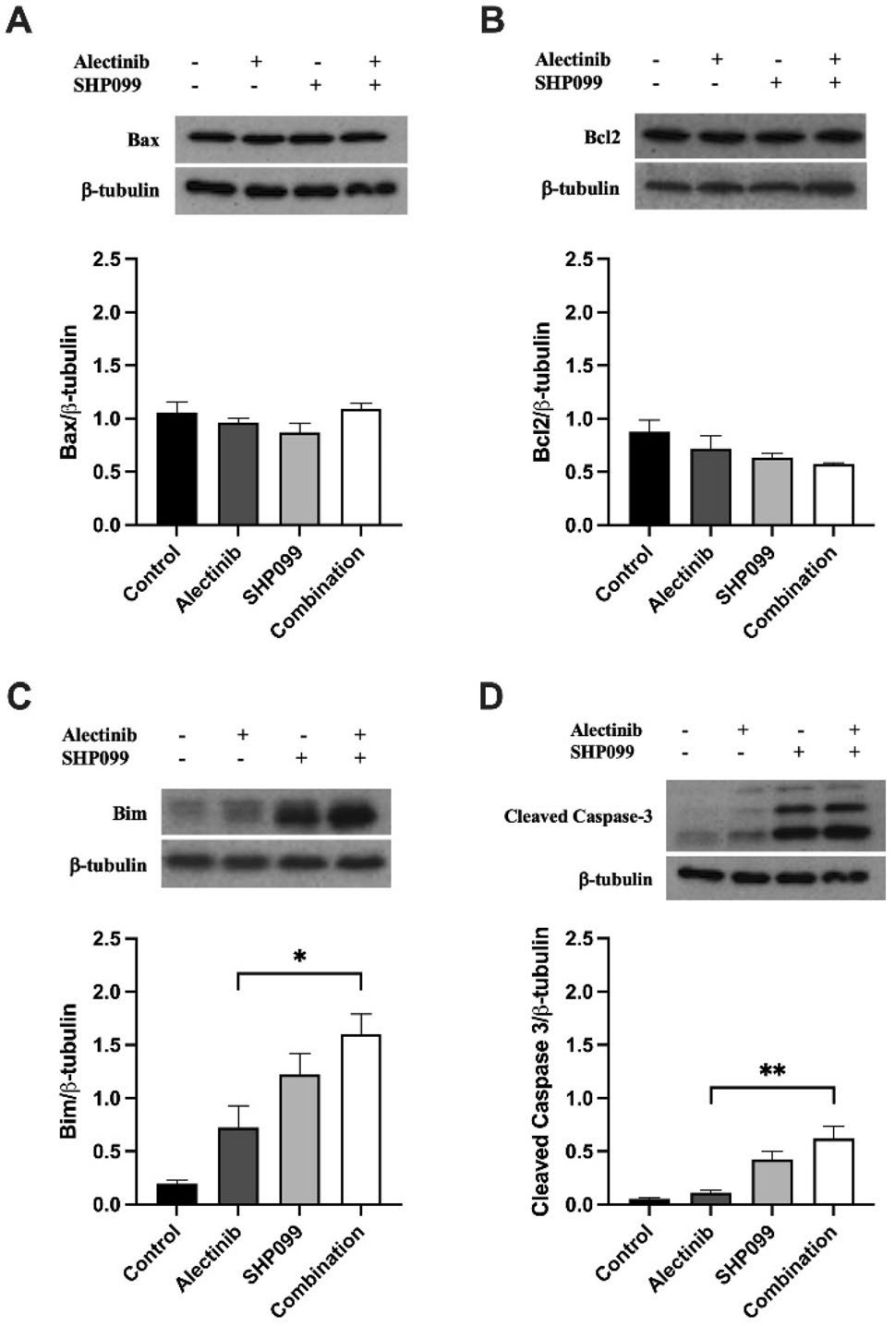
The effect of alectinib, SHP099, and the combination on apoptosis marker expression in H3122 cells. Representative and quantitative Western blots for (**a**) Bax, (**b**) Bcl2, (**c**) Bim, and (**d**) cleaved caspase-3, and the housekeeper protein β-tubulin after exposure to various drug treatments for 24 h. All data are expressed as mean ± SEM and were analysed using a one-way ANOVA followed by Bonferroni post hoc test. *represents a significant difference for alectinib vs combination, *p* < 0.05 and **represents a significant difference for alectinib vs combination, *p* < 0.01. n = 3 independent experiments.

**Figure 7:**
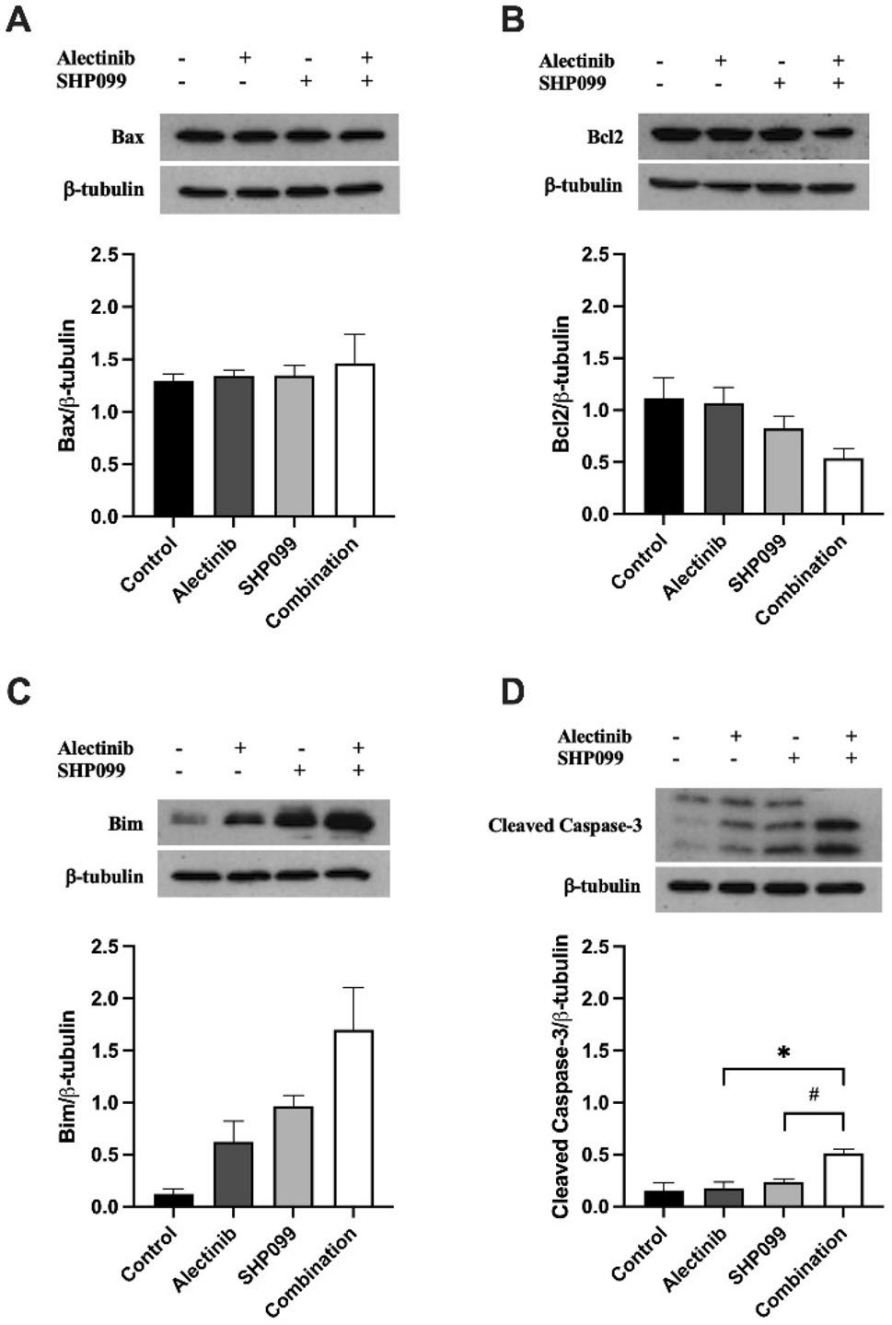
The effect of alectinib, SHP099, and the combination on apoptosis marker expression in H2228 cells. Representative and quantitative Western blots for (**a**) Bax, (**b**) Bcl2, (**c**) Bim, and (**d**) cleaved caspase-3, and the housekeeper protein β-tubulin after exposure to various drug treatments for 24 h. All data are expressed as mean ± SEM and were analysed using a one-way ANOVA followed by Bonferroni post hoc test. *represents a significant difference for alectinib vs combination, *p* < 0.05 and # represents a significant difference for SHP099 vs combination, *p* < 0.05. n = 3 independent experiments.

To characterise the effect of alectinib, SHP099, and the combination of alectinib/SHP099 on apoptosis in H3122 and H2228 cells we used Western blotting for protein mediators of apoptosis. Following 24-h exposure at cell viability EC_50_ concentrations (calculation details above), we measured changes in expression of the anti-apoptotic proteins, Bcl2 and Bax, and the pro-apoptotic protein, Bim, along with the marker of the *intrinsic* apoptosis pathway protease cleaved caspase-3. In both the H3122 and H2228 cell lines, there were no significant changes in either Bcl2 or Bax in any of the treatment groups (*p* > 0.05, Figs. 6A, B, 7A, B). By contrast, expression of Bim was very strongly increased by 5.3-fold and 7.2-fold following SHP099 alone or in combination with alectinib respectively, compared to the control in H3122 cells (*p* < 0.05, Fig. 6C). However, the presence of alectinib alone did not significantly increase Bim expression in H3122 cells compared to the control (*p* > 0.05, Fig. 6C) as did any of the drug treatments for H2228 cells (*p* > 0.05, Fig. 7C). Cleaved caspase-3 expression—a marker of the apoptosis cascade—was also distinctly increased by 6.9-fold and 0.5-fold respectively for H3122 and H2228 cells after 24 h in SHP099 monotherapy, in addition to a 10.6-fold and 2.3-fold increase for the combination compared to the control for H3122 and H2228 cells respectively (*p* < 0.05, Figs. 6D, 7D).

## Discussion

In this study, the effect of combining the SHP2 inhibitor, SHP099, with alectinib, an ALK inhibitor, was investigated in two different ALK^+^ NSCLC cell lines^22^. The results revealed that the combination of the two drugs resulted in synergistic suppression of cell growth compared to either drug alone. Additionally, Western blot experiments used to investigate the cellular mechanisms driving this synergistic effect demonstrated that the combination effect occurs in early (SHP2) and late (ERK) signalling steps for the major ALK pathway, with no combination effect on ALK activation. The combination effect is therefore an intermediary step between cytosolic ALK signalling and mediators of cell survival and proliferation, specifically in the regulation of the cell cycle and apoptosis; reflected in a continued, strong, combination/interaction effect for cell cycle mediators and mediators of the intrinsic apoptosis pathway.

We may conclude that the synergy of cancer cell growth suppression is apparent, from the impact of the potency of the combination of alectinib and SHP099 from the beginning of the ALK signalling pathway through multiple steps that encompass the regulation of the cell cycle and apoptosis. This suggests that patient trials co-titrating SHP2 inhibition with ALK inhibition may be a way to produce a specific inhibitory effect on cancer cell growth, whilst minimising toxicity in ALK-negative cells in the body. ALK may be therefore particularly suited for co-targeting with SHP2 in the treatment of NSCLC, as it is not widely expressed after embryonic development is complete^29^ (such that the systemic toxicity of ALK inhibitors is low).

Interestingly, although the effects of the drugs on cell viability were similar for H3122 and H2228 cell lines, the biochemical responses of the two cell lines were different in several ways, especially concerning the G2/M phase. Specifically, in H3122 cells, there was a significant decrease in cyclin B1 levels for alectinib alone compared to the combination, an effect not observed in the H2228 cells. This was similar for pCDK1, where in the H3122 cells there was a marked increase in the combination group compared to either drug alone but not in the H2228 cells. H3122 and H2228 cells harbour the EML4-ALK variant 1 and 3, respectively. Unlike variant 3, variant 1 retains the tandem atypical β-propeller (TAPE) in EML4. The TAPE domain has been reported to show greater sensitivity to ALK inhibitors, which could explain the differences in drug responses between the cell lines. In addition, H2228 cells have a significantly smaller proportion of WD40 repeats within TAPE in the EML4 domain compared to the H3122 cells^30^. WD40 is responsible for the organisation of mitotic spindles and hence mitotic progression^31^. Therefore, this could explain the differences in the cell cycle occurring between the cell lines, especially concerning the G2/M phase.

Previous work has demonstrated promising results for SHP099 in combination with ceritinib in resistant patient-derived cell lines, both in vitro and in vivo^32^. However, this current study is the first to demonstrate and measure the synergy of a strong and sustained combination effect from ALK through intracellular signalling to cell death. Our results support the hypothesis that the combination of SHP2 inhibition with ALK inhibition may have therapeutic potential in the treatment of ALK^+^ NSCLC by specifically targeting cancer cells. This is reflected in a strong, combination/interaction effect for cell cycle mediators and mediators of the intrinsic apoptosis pathway. In conclusion, these results suggest that SHP2 inhibition may be a way to improve outcomes with alectinib at sub-toxic doses by targeting the cancer cells while minimising toxicity in the rest of the body.

## Supplementary Information

Supplementary Information.

## Data Availability

The datasets generated during and/or analysed during the current study are available from the corresponding author on reasonable request.
