# Supplementary material for: Mechanisms of synergistic suppression of ALK-positive lung cancer cell growth by the combination of ALK and SHP2 inhibitors
Source: Sci Rep. 2023 Jun 20;13:10041. doi: 10.1038/s41598-023-37006-2 (PMC10281967; doi:10.1038/s41598-023-37006-2)
Supplement: Supplementary file 1 — Supplementary Information. [file 41598_2023_37006_MOESM1_ESM.pdf]

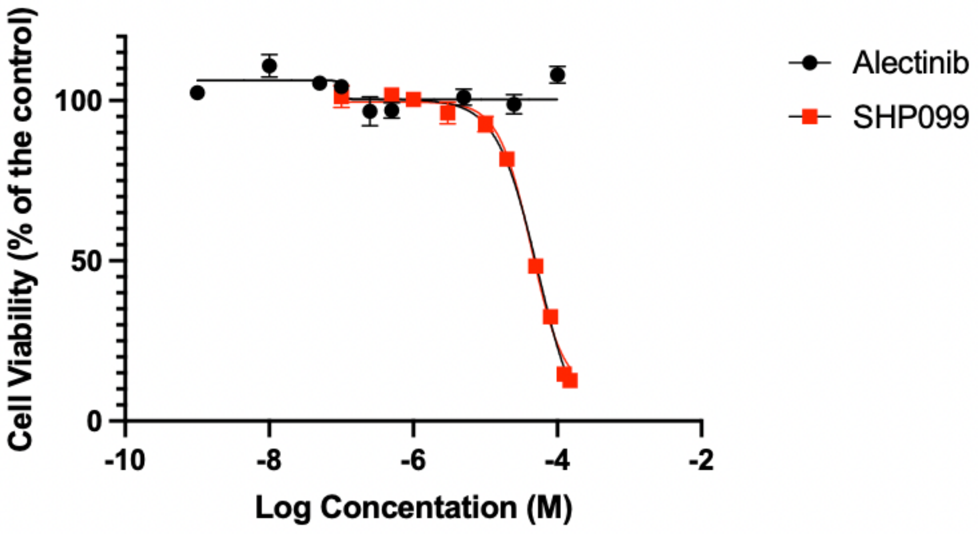

Figure S1. The effect of the ALK inhibitor alectinib and the SHP2 inhibitor SHP099 on cell viability of A549 cells. Data is expressed as mean ± SEM.

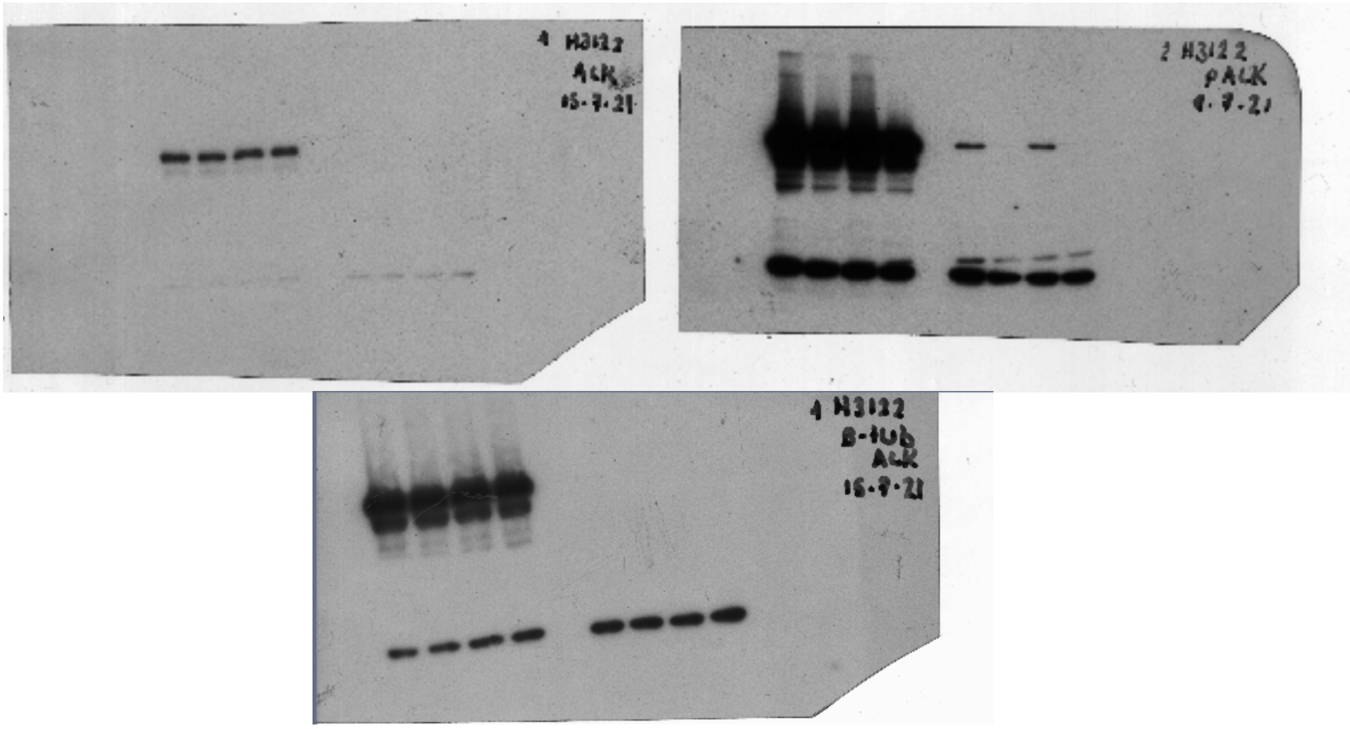

Figure S2. Raw image of Western blots for ALK, pALK and β-tubulin in H3122 cells used in figure 3A.

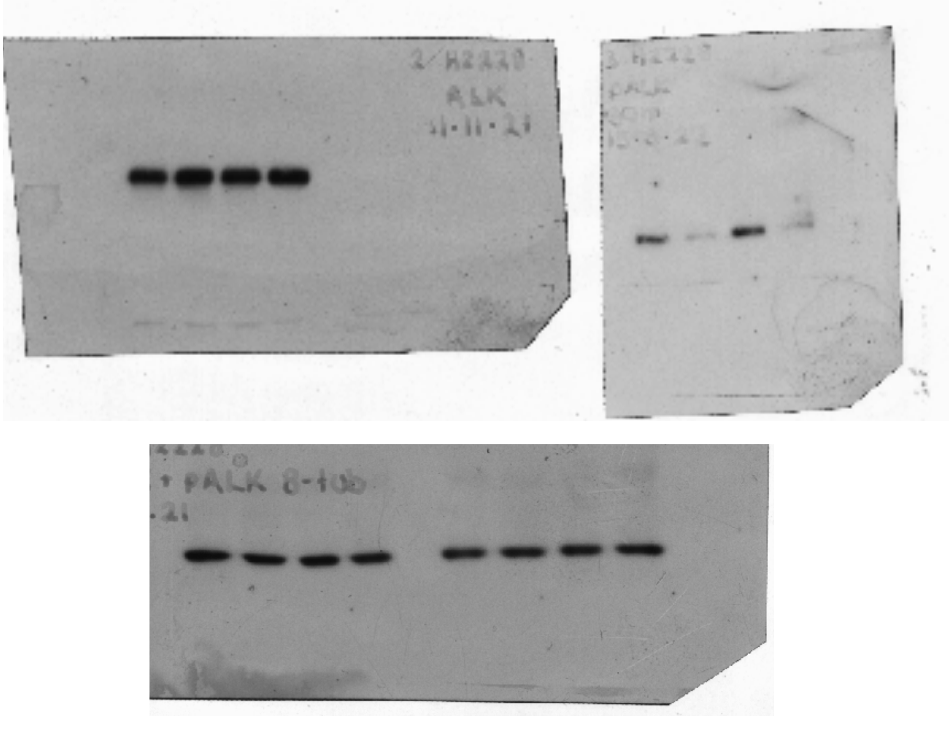

Figure S3. Raw image of Western blots for ALK, pALK and β-tubulin in H2228 cells used in figure 3D.

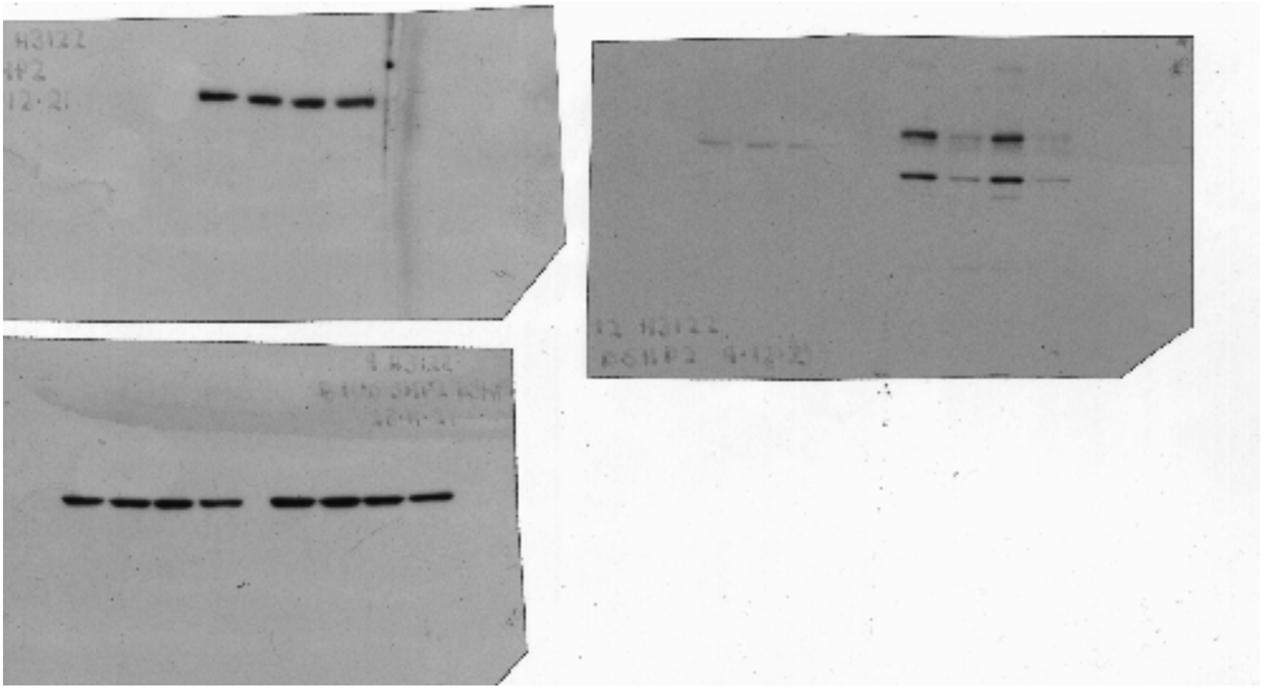

Figure S4. Raw image of Western blots for SHP2, pSHP2 and β-tubulin in H3122 cells used in figure 3B.

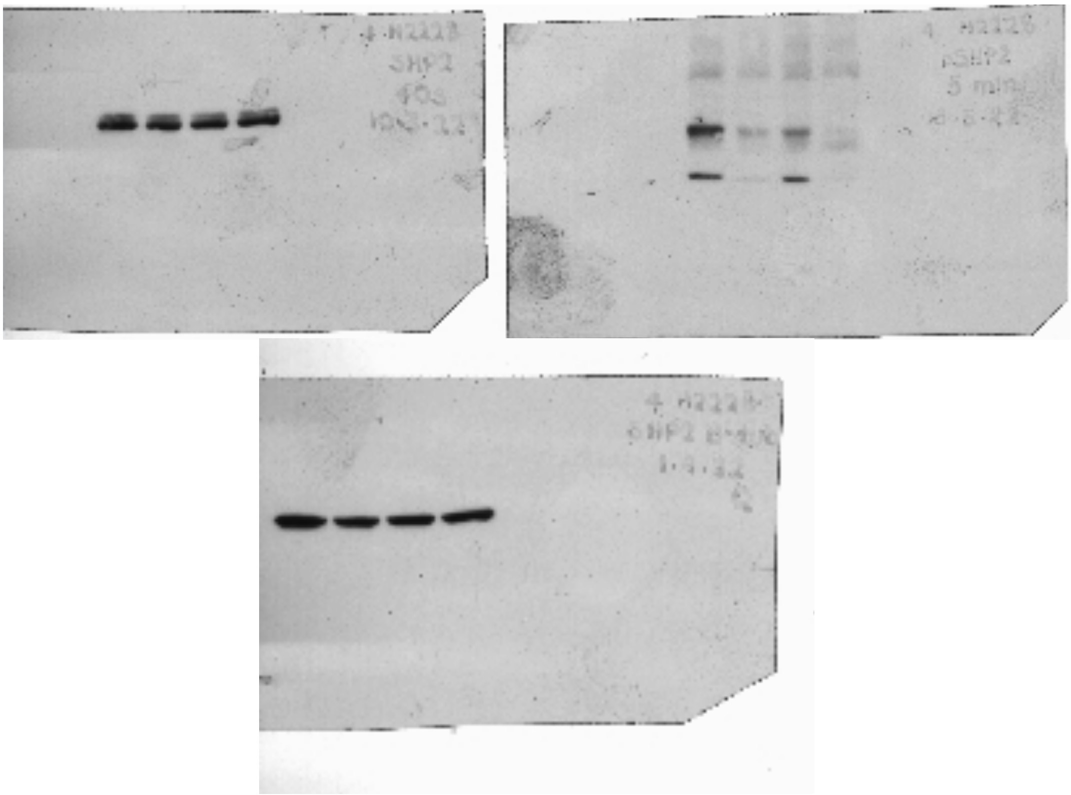

Figure S5. Raw image of Western blots for SHP2, pSHP2 and β-tubulin in H2228 cells used in figure 3E.

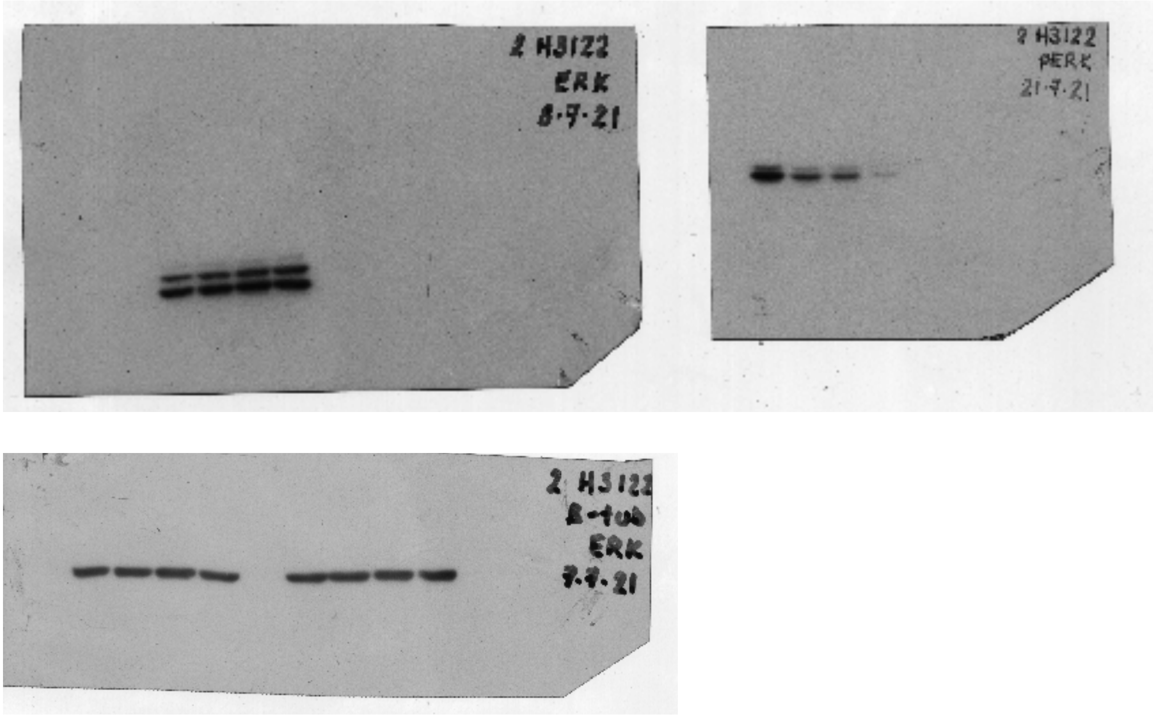

Figure S6. Raw image of Western blots for ERK, pERK and β-tubulin in H3122 cells used in figure 3C.

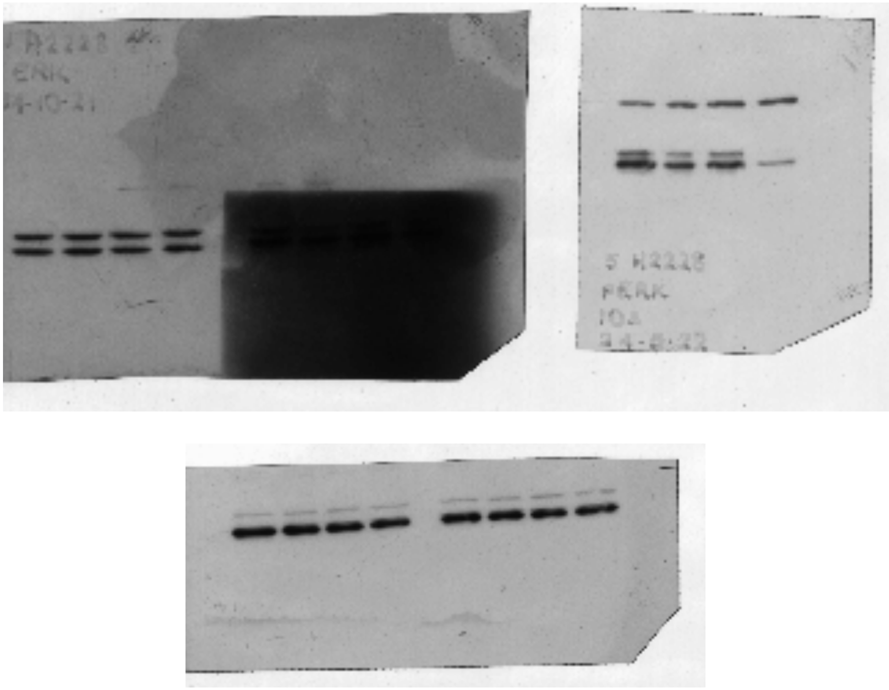

Figure S7. Raw image of Western blots for ERK, pERK and β-tubulin in H2228 cells used in figure 3F.

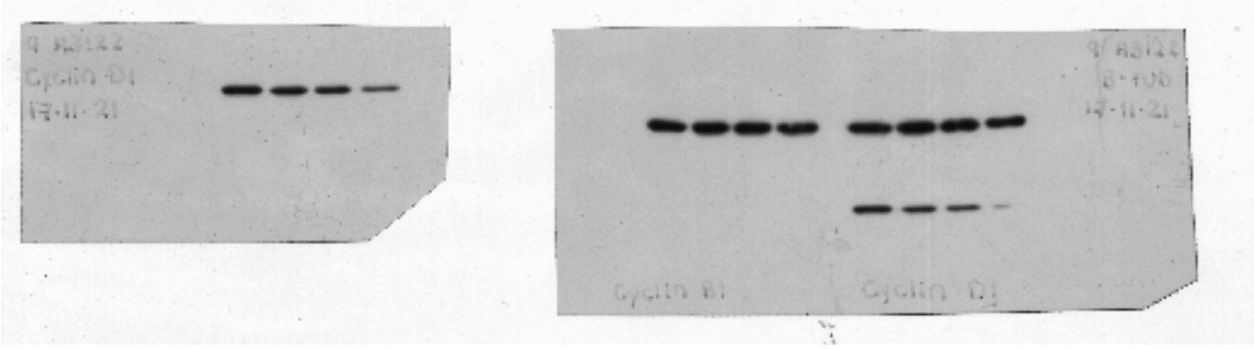

Figure S8. Raw image of Western blots for Cyclin D1 and β-tubulin in H3122 cells used in figure 5A.

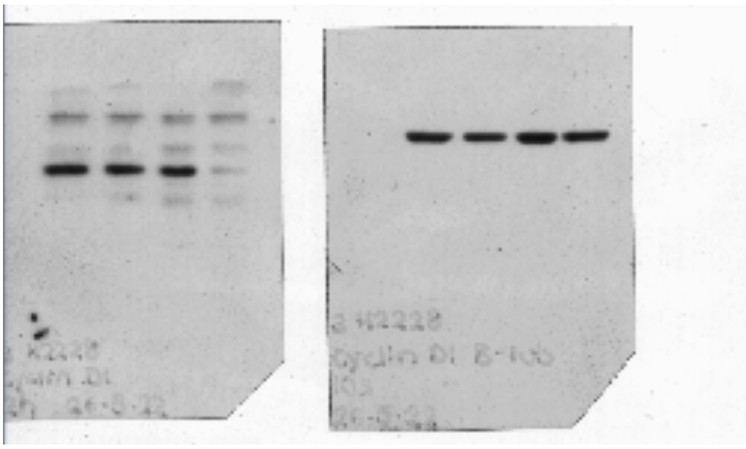

Figure S9. Raw image of Western blots for Cyclin D1 and β-tubulin in H2228 cells used in figure 6A.

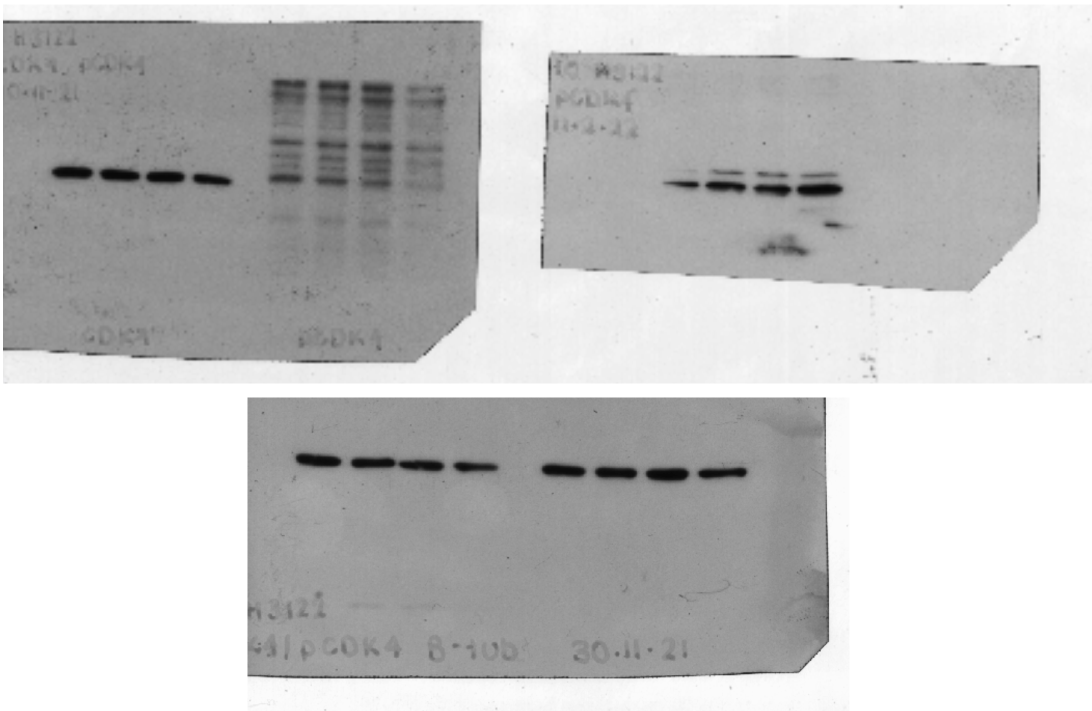

Figure S10. Raw image of Western blots for CDK4, pCDK4 and β-tubulin in H3122 cells used in figure 5B.

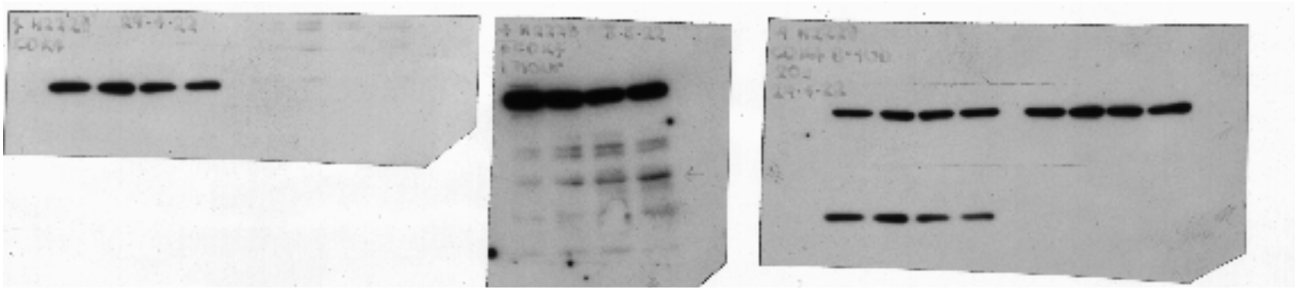

Figure S11. Raw image of Western blots for CDK4, pCDK4 and β-tubulin in H2228 cells used in figure 6B.

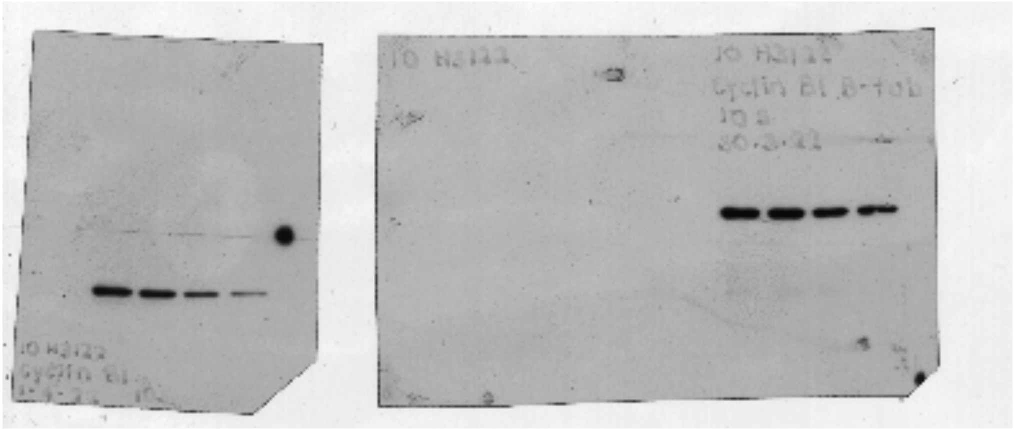

Figure S12. Raw image of Western blots for Cyclin B1 and β-tubulin in H3122 cells used in figure 5C.

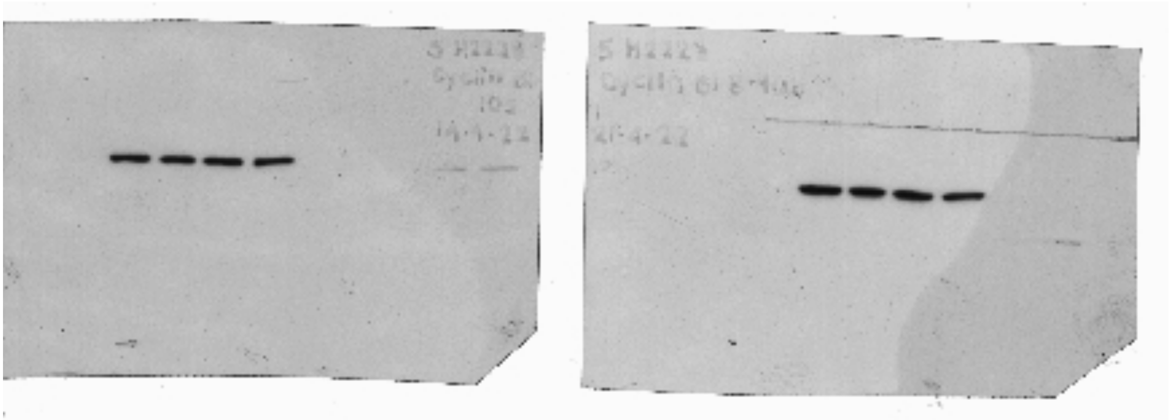

Figure S13. Raw image of Western blots for Cyclin B1 and β-tubulin in H2228 cells used in figure 6C.

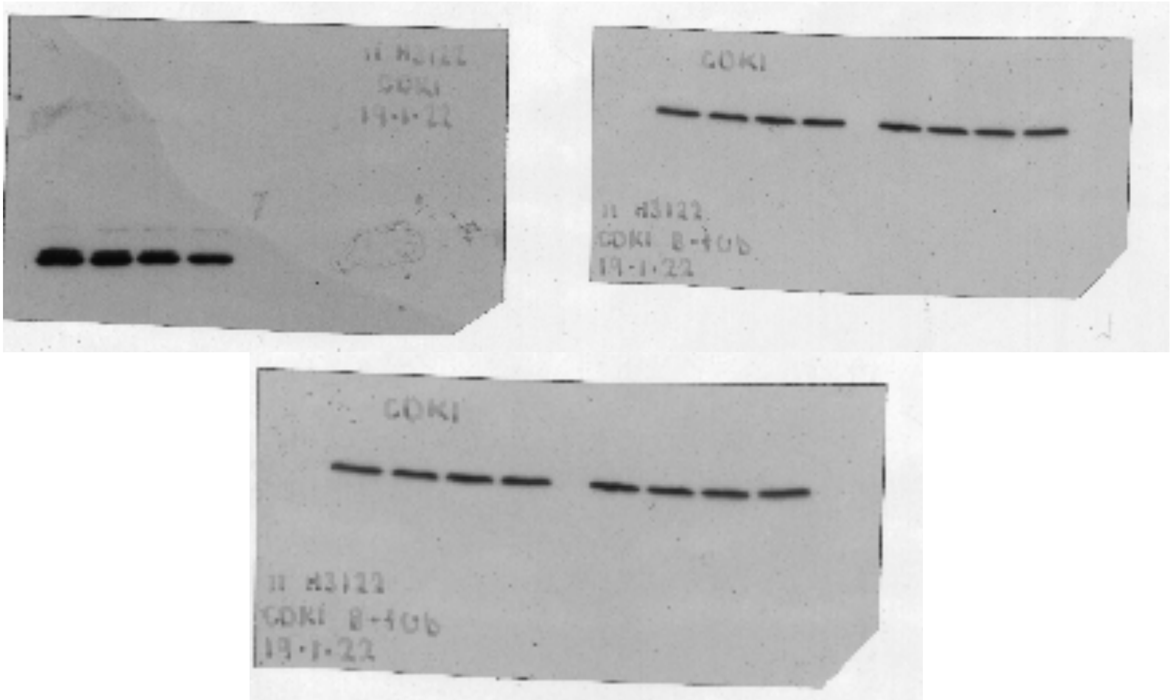

Figure S14. Raw image of Western blots for CDK1, pCDK1 and β-tubulin in H3122 cells used in figure 5D.

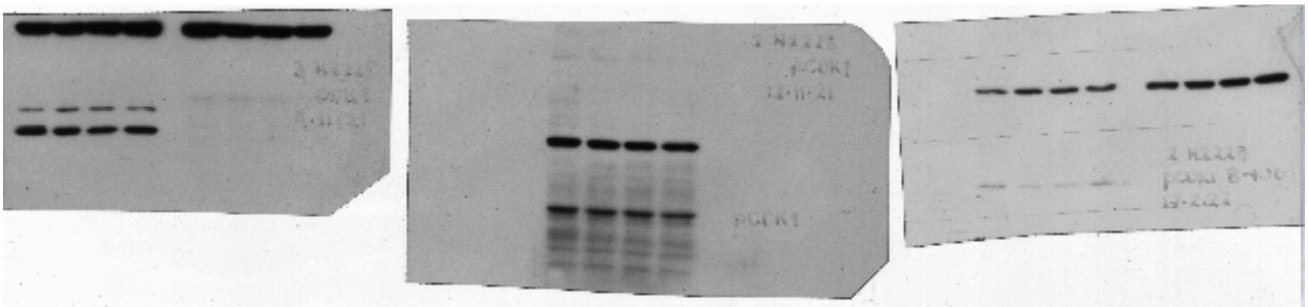

Figure S15. Raw image of Western blots for CDK1, pCDK1 and β-tubulin in H2228 cells used in figure 6D.

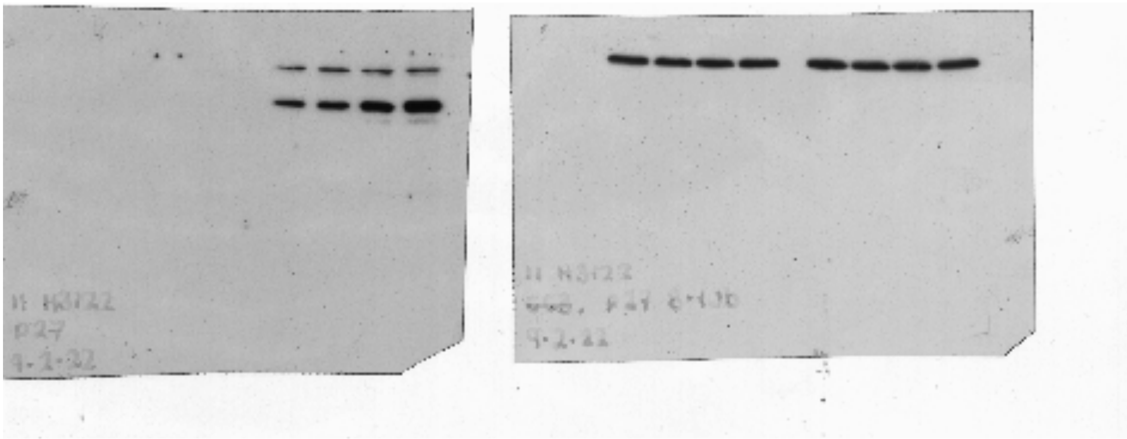

Figure S16. Raw image of Western blots for p27 and β-tubulin in H3122 cells used in figure 5E.

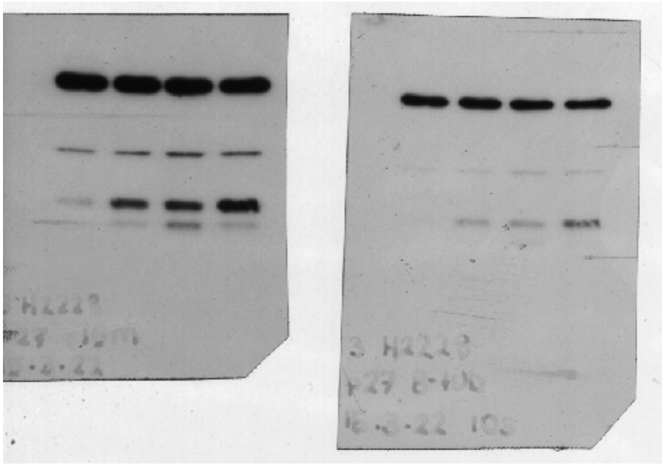

Figure S17. Raw image of Western blots for p27 and β-tubulin in H2228 cells used in figure 6E.

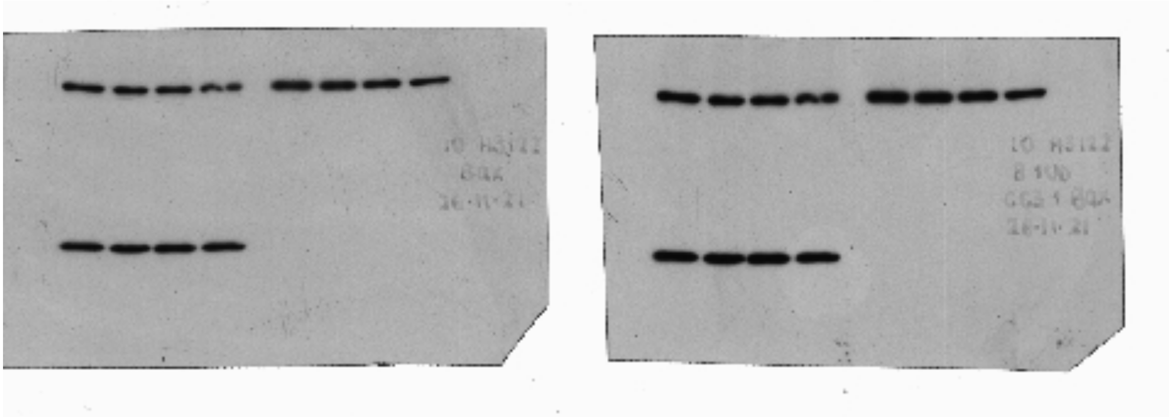

Figure S18. Raw image of Western blots for Bax and β-tubulin in H3122 cells used in figure 7A.

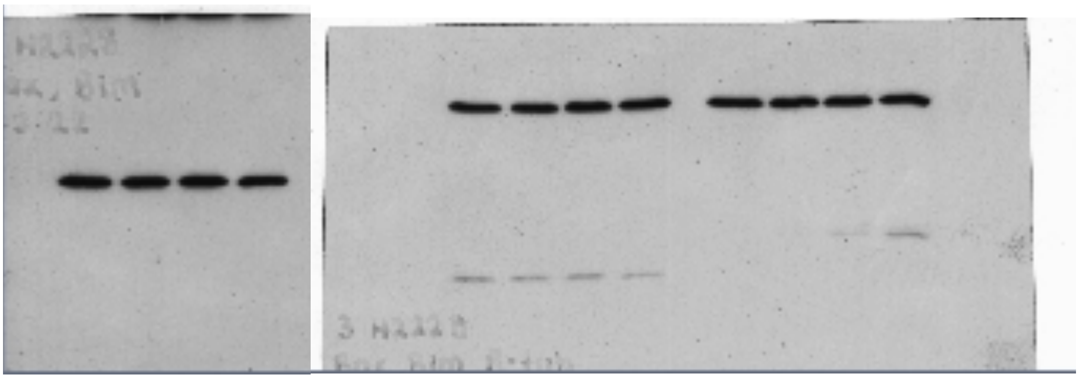

Figure S19. Raw image of Western blots for Bax and β-tubulin in H2228 cells used in figure 8A.

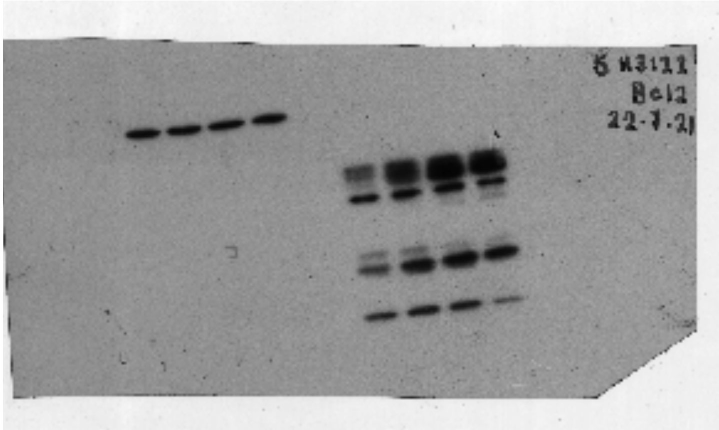

Figure S20. Raw image of Western blots for Bcl2 and β-tubulin in H3122 cells used in figure 7B.

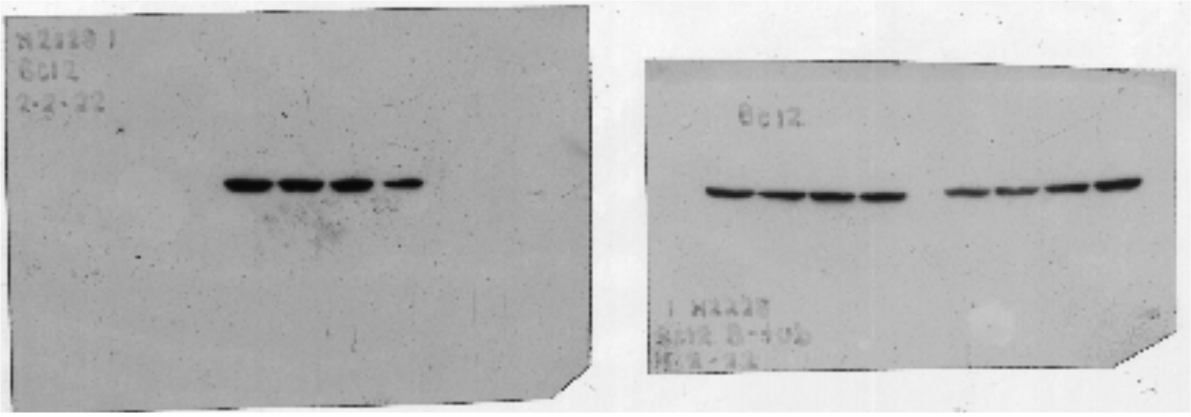

Figure S21. Raw image of Western blots for Bcl2 and β-tubulin in H2228 cells used in figure 8B.

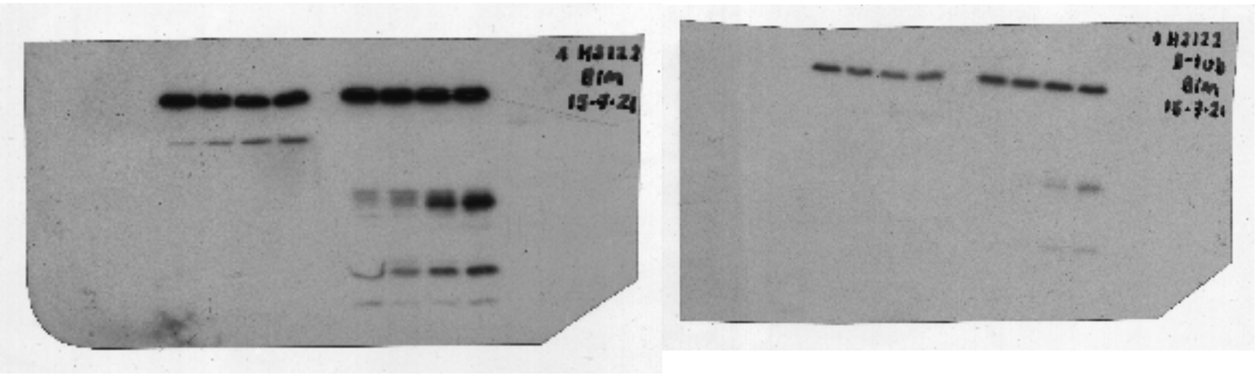

Figure S22. Raw image of Western blots for Bim and β-tubulin in H3122 cells used in figure 7C.

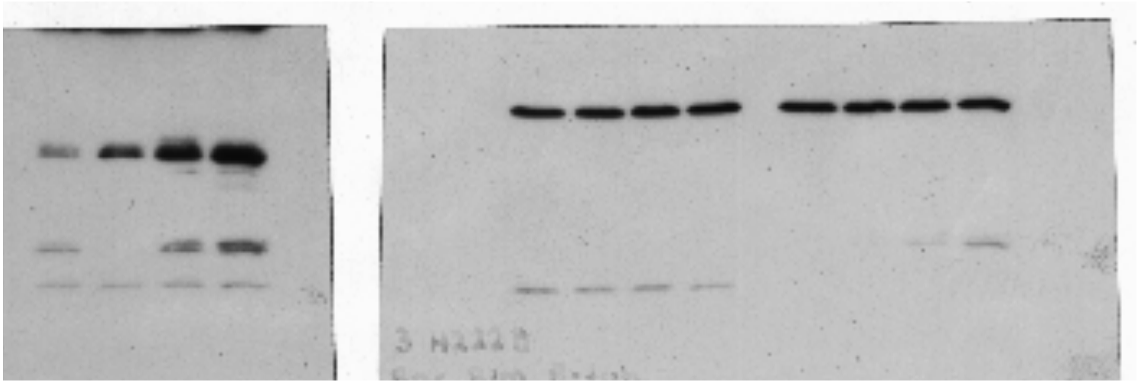

Figure S23. Raw image of Western blots for Bim and β-tubulin in H2228 cells used in figure 8C.

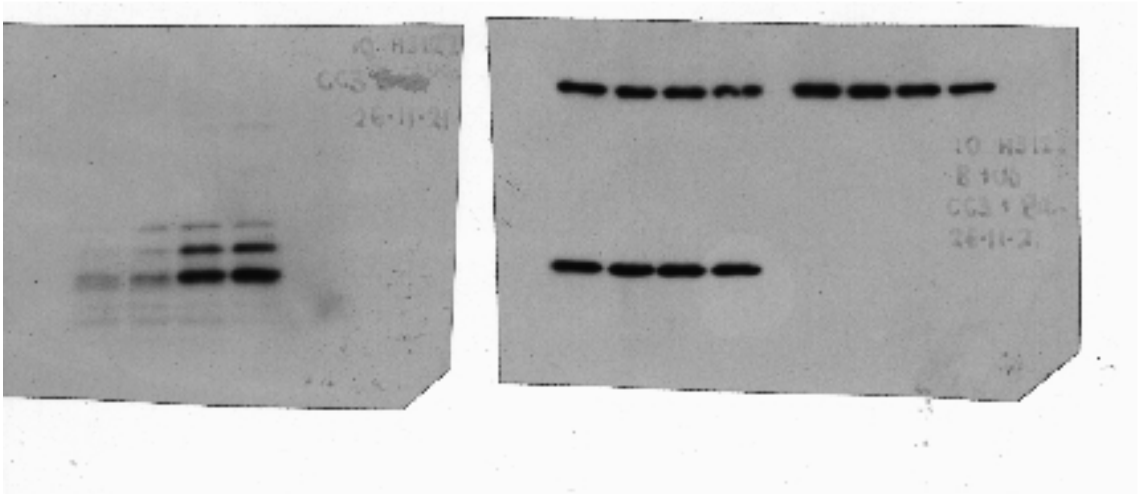

Figure S24. Raw image of Western blots for Cleaved caspase 3 and β-tubulin in H3122 cells used in figure 7D.

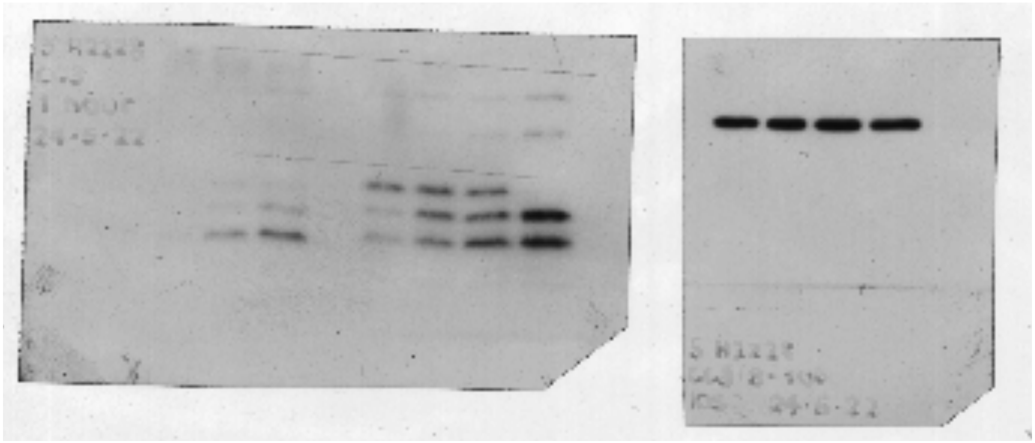

Figure S25. Raw image of Western blots for Cleaved caspase 3 and β-tubulin in H2228 cells used in figure 8D.
